# Supplementary material for: Is it possible to compare inhibitory and excitatory intracortical circuits in face and hand primary motor cortex?
Source: J Physiol. 2022 Jul 14;600(15):3567–83. doi: 10.1113/JP283137 (PMC9544430; doi:10.1113/JP283137)
Supplement: Supplementary file 2 — Statistical Summary Document [file TJP-600-3567-s002.pdf]

The Journal of Physiology

Statistical Summary Document

Manuscript Title: Inhibitory and excitatory intracortical circuits function differently in face and hand primary motor cortex.

Authors: Francesca Ginatempo, Nicola Loi, Andrea Manca, John C. Rothwel, Franca Deriu,

Animal model used, if applicable: No applicable.

Underlying hypothesis: Different role of inhibitory and facilitatory intracortical circuits in the motor control of face and hand muscles.

Definitions of ‘n’:

Question 1: n =15. Short-interval intracortical inhibition (SICI) of M1 innervating the DAO and FDI muscles at rest.
Question 2: n = 15 Short-interval intracortical inhibition (SICI) of M1 innervating the DAO and FDI muscles during voluntary muscle contraction.
Question 3: n = 15 Short-interval intracortical facilitation (SICF) of M1 innervating the DAO and FDI muscles at rest.
Question 4: n = 15 Short-interval intracortical facilitation (SICF) of M1 innervating the DAO and FDI muscles during voluntary muscle contraction.
Question 5: n = 14 Cortical silent period of the M1 innervating DAO and FDI muscles.

Statistical summary table:

Experimental question number*	Finding/ conclusion	Experimental location/ variable<br>e.g. muscle, neocortex or genotype	Mean value<br>(or other summary statistic)	SD	n val.	P**	Units	Data comparisons<br>e.g. WT vs KO	Statistical test	Any other variable<br>e.g. subjects’ age or sex	Figure/ table in which data are presented	Comments<br>e.g. observation
1. To investigate Short-interval intracortical inhibition (SICI) of M1 innervating the DAO and FDI muscles at rest.	In the resting DAO, a significant inhibition was observed at subthreshold CS intensities such as 60%-80% RMT.<br>In the resting FDI, a	TMS of the facial and hand motor cortex with recordings from the DAO and FDI muscle respectively: the main experimental (independent)	See table 1 below	See table 1 below	15	**FACE-M1**<br>TS vs 1.0 ms: p=0.003;<br>TS vs 2.0: p=0.007;<br>TS vs 3.0 ms: p=0.005).<br>1.0 ms -60%	MEP amplitude: mV	Unconditioned MEP versus conditioned MEP<br>MEP ratio DAO versus	ANOVA and Bonferroni-corrected post-hoc t-tests	15 healthy volunteers (10 females and 5 males; mean age 28.40 (6.31) years)	Figure 1 and 3 (A and B)	

	significant inhibition was observed at subthreshold and threshold CS intensities 50-100% RMT for all the ISIs studied. At rest, SICI was stronger in FDI than DAO	variable is the motor evoked potential (MEP) amplitude and the ratio between the amplitude of conditioned MEP and unconditioned MEPs<br><br>TMS protocol with a subthreshold conditioning stimulus (CS) preceding a suprathreshold TS by an ISI of 1.0, 2.0 and 3.0 ms. The CS intensity was set between 50 and 100% of RMT, in steps of 10%, and the TS intensity at 120% of RMT				p=0.001;<br><br>3.0 ms-60% p=0.028,<br><br>1.0 ms -70% p=0.01,<br><br>3.0 ms -70% p=0.012,<br><br>1.0 ms -80% p=0.022,<br><br>3.0 ms -80% p=0.004<br><br>2.0 ms - 60% p=0.010<br><br>2.0 ms - 80% p=0.009<br><br>**HAND-M1**<br><br>CS intensities of 1.0 ms -60% p=0.003;<br><br>2.0 ms-60%: p=0.008;<br><br>3.0 ms-60%: p=0.019,<br><br>1.0 ms -70% p=0.0016;<br><br>3.0 ms-70%: p=0.027		MEP ratio FDI				

						1.0 ms -80% p=0.004;<br>2.0 ms-80%: p=0.015;<br>3.0 ms-80%: p=0.005,<br>1.0 ms -90% p=0.003;<br>2.0 ms-90%: p=0.006;<br>3.0 ms-90%: p=0.024,<br>2.0 ms-100%: p=0.015;						
2. To investigate Short-interval intracortical inhibition (SICI) of M1 innervating the DAO and FDI muscles during voluntary muscle contraction.	In the active DAO, a clear SICI was observed for all the ISIs studied but only with a subthreshold CS intensity such as of 80% AMT.<br>In the active FDI, a weak SICI was found.<br>In the active	TMS of the facial and hand motor cortex with recordings from the DAO and FDI muscle respectively: the main experimental (independent) variable is the motor evoked potential (MEP) amplitude and the ratio	See table 2 below	See table 2 below	15	**FACE- M1:**<br>1.0 ms - 50% p=0.022<br>1.0 ms- 70% p=0.001<br>1.0 ms- 80% p=0.016<br>2.0 ms- 80% p=0.009,<br>3.0 ms- 80% p=0.041	MEP amplitude: mV	Unconditioned MEP versus conditioned MEP<br>MEP ratio DAO versus MEP ratio FDI	ANOVA and Bonferroni-corrected post-hoc t-tests	15 healthy volunteers (10 females and 5 males; mean age 28.40 (6.31) years)	Figure 2 and 3 (C and D)	

	muscle condition, SICI was weaker in FDI than DAO	between the amplitude of conditioned MEP and unconditioned MEPs<br><br>Active SICI was studied during isometric contraction of the tested muscle at 10% of MVIC from the right DAO and FDI muscles. Active SICI was elicited using a paired-pulse TMS protocol with a subthreshold CS preceding a suprathreshold TS by an ISI of 1.0, 2.0 and 3.0 ms. The CS intensity was set between 50 and 100% of AMT, in steps of 10%, and the TS intensity at										

The Journal of Physiology Statistical Summary Document

		120% of AMT.										
3. To investigate Short-interval intracortical facilitation (SICF) of M1 innervating the DAO and FDI muscles at rest.	In the relaxed DAO, a clear facilitation was observed at threshold and suprathreshold CS intensities only at shortes ISIs such as at 1.0 and 1.5 ms ISIs. In the relaxed FDI, a clear SICF was observed with threshold and suprathreshold CS intensities at ISIs of 1.0-1.5 ms and 2.5-3.0 ms.<br><br>SICF at rest was similar in FDI and DAO muscles.	TMS of the facial and hand motor cortex with recordings from the DAO and FDI muscle respectively: the main experimental (independent) variable is the motor evoked potential (MEP) amplitude and the ratio between the amplitude of conditioned MEP and unconditioned MEPs<br><br>Rest SICF was elicited using a paired-pulse TMS protocol with a sub- and suprathreshold CS succeeding a suprathreshold	See table 3 below	See table 3 below	15	**FACE-M1**<br><br>2.5 ms- 100% p=0.029<br><br>1.0 ms- 110% p=0.029<br><br>**HAND-M1**<br><br>1.0ms-90% p=0.053<br><br>1.5 ms-90% p=0.029<br><br>1.0ms-100% p=0.051<br><br>1.5 ms-100% p=0.037, respectively)<br><br>1.0ms-110% p=0.031<br><br>1.5 ms-110% p=0.015<br><br>3.0 ms-100% p=0.018<br><br>2.5 ms-100% p=0.030	MEP amplitude: mV	Unconditioned MEP versus conditioned MEP<br><br>MEP ratio DAO versus MEP ratio FDI	ANOVA and Bonferroni-corrected post-hoc t-tests	15 healthy volunteers (10 females and 5 males; mean age 28.40 (6.31) years)	Figure 4 and 6 (A and B)	

		TS by ISIs of 1.0, 1.5, 2.0, 2.5, 3.0 and 3.5 ms. The CS intensity was set between 80 and 110% of RMT, in steps of 10%, and the TS intensity at 120% of RMT										
4. To investigate Short-interval intracortical facilitation (SICF) of M1 innervating the DAO and FDI muscles during voluntary muscle contraction.	A clear facilitation was observed at 1.0 and 1.5 ISIs at both subthreshold and threshold CS intensities.<br>A clear SICF was detected at 1.5 ms with 100 and 110% AMT.<br>In the active muscle condition, SICF was stronger in FDI than DAO.	TMS of the facial and hand motor cortex with recordings from the DAO and FDI muscle respectively: the main experimental (independent) variable is the motor evoked potential (MEP) amplitude and the ratio between the amplitude of conditioned MEP and	See table 4 below	See table 4 below	15	**FACE-M1**<br>TS versus 1.0 ms p=0.038,<br>TS versus 1.5 ms p=0.032<br>TS versus 3.0 ms (p=0.027)<br>HAND-M1<br>1.5 ms - 100% p=0.04)<br>1.5 ms - 110% p=0.035<br>3.5 ms- 110% p=0.041	MEP amplitude: mV	Unconditioned MEP versus conditioned MEP<br>MEP ratio DAO versus MEP ratio FDI	ANOVA and Bonferroni-corrected post-hoc t-tests	15 healthy volunteers (10 females and 5 males; mean age 28.40 (6.31) years)	Figure 5 and 6 (C and D)	

		unconditioned MEPs<br>Active SICF was studied during isometric contraction of the tested muscle at 10% of MVIC. Active SICF was elicited using a paired-pulse TMS protocol with a sub- and suprathreshold CS succeeding a suprathreshold TS by ISIs of 1.0, 1.5, 2.0, 2.5, 3.0 and 3.5 ms. The CS intensity was set between 80 and 110% of AMT and the TS intensity at 120% of AMT.									
5. To investigate Cortical silent period of the M1	The CSP was not different between DAO and FDI	The cortical silent period (CSP) from the right DAO	See table 5 below	See table 5 below	14	140% AMT versus 130% AMT (p<0.001)	Duration of Cortical silent period: ms	CSP 120%, 130 and	ANOVA and Bonferroni-corrected post-hoc t-tests	14 healthy volunteers (9 females and 5 males; mean age	ANOVA and Bonferroni-corrected	

innervating DAO and FDI muscles.		and FDI muscles using a single pulse stimulus at an intensity of 120, 130 and 140% AMT. The CSP was recorded in two different conditions: CSP 10% and CSP 100%, during the isometric contraction of the tested muscle at 10% and 100% of MVIC.				140% AMT versus 120% AMT (p<0.001) in both muscles.		140% AMT and during a voluntary contraction of 10% and 100% of MVIC.		28.30 (6.21) years)	post-hoc t-tests	

*You may use multiple lines for the same question to indicate multiple comparisons

** Authors may wish to make the text bold where p is considered significant against a stated confidence limit.

The Journal of Physiology

Statistical Summary Document

Table 1: data are expressed as mean ± Standard deviation. Motor evoked potential, MEP; Depressor Anguli oris, DAO; First dorsalis interosseus, FDI; resting motor threshold, RMT.

**MEP Amplitude DAO muscle**	**TEST MEP**	**1.0 ms**	**2.0 ms**	**3.0 ms**
50% RMT	0.18±0.13	0.10±0.06	0.15±0.11	0.15±0.11
60% RMT	0.16±0.08	0.07±0.02	0.11±0.06	0.11±0.06
70% RMT	0.16±0.10	0.08±0.03	0.12±0.08	0.09±0.05
80% RMT	0.17±0.10	0.11±0.05	0.11±0.07	0.09±0.04
90% RMT	0.22±0.15	0.20±0.16	0.15±0.12	0.18±0.12
100% RMT	0.22±0.15	0.28±0.25	0.19±0.16	0.21±0.14
**MEP Amplitude FDI muscle**	**TEST MEP**	**1.0 ms**	**2.0 ms**	**3.0 ms**
50% RMT	1.38±1.32	1.01±0.91	1.29±1.14	1.27±0.94
60% RMT	1.51±1.13	0.58±0.84	1.11±1.11	0.92±0.94
70% RMT	1.73±1.82	0.42±0.53	1.08±1.52	0.96±1.38
80% RMT	1.93±1.73	0.60±0.89	0.85±0.97	0.52±0.50
90% RMT	1.65±1.50	0.92±0.99	0.91±1.21	0.94±0.99
100% RMT	2.09±2.04	1.53±1.63	1.04±1.25	1.58±1.55

**MEP Amplitude Ratio DAO muscle**	**1.0 ms**	**2.0 ms**	**3.0 ms**
50% RMT	0.66±0.19	0.90±0.47	0.91±0.28
60% RMT	0.49±0.19	0.71±0.33	0.70±0.23
70% RMT	0.55±0.25	0.78±0.33	0.61±0.25
80% RMT	0.66±0.24	0.67±0.18	0.56±0.19
90% RMT	0.85±0.29	0.69±0.27	0.82±0.23
100% RMT	1.20±0.31	0.85±0.31	1.01±0.31
**MEP Amplitude Ratio FDI muscle**	**1.0 ms**	**2.0 ms**	**3.0 ms**
50% RMT	0.91±0.52	1.11±0.64	1.13±0.52
60% RMT	0.39±0.35	0.75±0.47	0.62±0.33
70% RMT	0.36±0.34	0.66±0.50	0.50±0.36
80% RMT	0.30±0.19	0.52±0.41	0.30±0.14
90% RMT	0.50±0.22	0.47±0.31	0.63±0.76
100% RMT	0.75±0.37	0.60±0.58	0.82±0.33

Table 2: data are expressed as mean ± Standard deviation. Motor evoked potential, MEP; Depressor Anguli oris, DAO; First dorsalis interosseus, FDI; resting motor threshold, RMT.				
**MEP AMplitude DAO muscles**	**TEST MEP**	**1.0 ms**	**2.0 ms**	**3.0 ms**
50% AMT	0.52±0.50	0.43±0.47	0.50±0.55	0.46±0.44
60% AMT	0.51±0.50	0.37±0.33	0.47±0.42	0.48±0.41
70% AMT	0.59±0.44	0.40±0.33	0.52±0.43	0.50±0.49
80% AMT	0.69±0.61	0.49±0.42	0.59±0.56	0.45±0.38
90% AMT	0.66±0.55	0.56±0.43	0.66±0.52	0.48±0.32
100%AMT	0.69±0.52	0.71±0.53	0.72±0.59	0.63±0.48
**MEP AMplitude FDI muscles**	**TEST MEP**	**1.0 ms**	**2.0 ms**	**3.0 ms**
50% AMT	1.26±0.64	1.10±0.71	1.20±0.72	1.17±0.67
60% AMT	1.14±0.91	1.09±0.94	1.22±1.0	1.02±0.87
70% AMT	1.13±0.60	0.99±0.59	1.30±0.99	1.18±0.82
80% AMT	1.24±0.83	0.84±0.55	1.39±1.15	1.120±0.77
90% AMT	1.44±0.85	1.04±0.59	1.49±1.06	1.22±0.81
100%AMT	1.40±0.75	1.66±1.37	1.88±1.62	1.45±0.77
**MEP Amplitude Ratio DAO muscle**	**1.0 ms**	**2.0 ms**	**3.0 ms**	
50% AMT	0.81±0.21	0.92±0.17	0.91±0.23	
60% AMT	0.78±0.19	0.99±0.22	1.00±0.24	
70% AMT	0.64±0.12	0.87±0.20	0.80±0.23	
80% AMT	0.76±0.14	0.88±0.17	0.72±0.23	
90% AMT	0.88±0.18	1.05±0.29	0.79±0.20	
100%AMT	1.03±0.25	1.04±0.26	0.90±0.17	
**MEP Amplitude Ratio FDI muscle**	**1.0 ms**	**2.0 ms**	**3.0 ms**	
50% AMT	0.87±0.30	0.95±0.22	0.92±0.21	
60% AMT	0.96±0.26	1.07±0.20	0.89±0.16	
70% AMT	0.91±0.27	1.08±0.37	1.01±0.29	
80% AMT	0.76±0.30	1.05±0.29	0.99±0.33	
90% AMT	0.78±0.25	1.04±0.38	0.89±0.34	
100%AMT	1.12±0.48	1.26±0.67	1.07±0.44	

Table 3: data are expressed as mean ± Standard deviation. Motor evoked potential, MEP; Depressor Anguli oris, DAO; First dorsalis interosseus, FDI; resting motor threshold, RMT.

**MEP Amplitude DAO muscle**	**TEST MEP**	**1.0 ms**	**1.5 ms**	**2.0 ms**	**2.5 ms**	**3.0 ms**	**3.5 ms**
80% RMT	0.21 ±0.24	0.27±0.35	0.33±0.42	0.26±0.32	0.28±0.34	0.25±0.28	0.25±0.38
90% RMT	0.18 ±0.24	0.37±0.32	0.28±0.26	0.28±0.27	0.28±0.29	0.24±0.22	0.23±0.16
100% RMT	0.23±0.16	0.41±0.39	0.47±0.43	0.33±0.32	0.40±0.31	0.39±0.37	0.27±0.19
110% RMT	0.27±0.33	0.44±0.45	0.52±0.51	0.38±0.41	0.51±0.52	0.47±0.49	0.38±0.46
**MEP Amplitude FDI muscle**	**TEST MEP**	**1.0 ms**	**1.5 ms**	**2.0 ms**	**2.5 ms**	**3.0 ms**	**3.5 ms**
80% RMT	1.63±0.1.06	2.18±1.62	2.39±1.61	1.62±1.13	2.19±1.46	2.12±1.66	1.90±1.58
90% RMT	1.66 ±1.44	2.32±1.67	2.42±1.75	1.85±1.49	2.17±1.74	2.30±1.63	1.71±1.23
100% RMT	1.90±1.34	2.78±1.77	3.05±2.02	2.09±1.76	2.91±2.04	2.82±1.90	2.18±1.57
110% RMT	1.96±1.41	3.02±2.00	3.16±1.79	2.03±1.73	3.11±1.81	2.83±1.82	2.00±1.34

**MEP Amplitude Ratio DAO muscle**	**1.0 ms**	**1.5 ms**	**2.0 ms**	**2.5 ms**	**3.0 ms**	**3.5 ms**
80% RMT	1.27±0.47	1.47±0.53	1.27±0.48	1.32±0.45	1.26±0.31	1.07±0.32
90% RMT	1.52±0.41	1.92±0.53	1.50±0.55	1.52±0.38	1.41±0.62	1.32±0.42
100% RMT	1.75±0.67	1.97±0.71	1.50±0.60	1.84±0.62	1.71±0.65	1.27±0.52
110% RMT	1.84±0.70	2.22±1.09	1.57±0.62	2.08±1.03	1.97±1.0	1.52±0.56
**MEP Amplitude Ratio FDI muscle**	**1.0 ms**	**1.5 ms**	**2.0 ms**	**2.5 ms**	**3.0 ms**	**3.5 ms**
80% RMT	1.34±0.43	1.61±0.65	1.14±0.54	1.34±0.27	1.35±0.51	1.10±0.28
90% RMT	1.50±0.53	1.57±0.51	1.20±0.46	1.32±0.49	1.50±0.56	1.17±0.43
100% RMT	1.64±0.60	1.79±0.82	1.11±0.34	1.55±0.56	1.53±0.53	1.18±0.42
110% RMT	1.67±0.60	1.86±0.79	1.04±0.42	1.80±0.93	1.55±0.60	1.06±0.43

Table 4: data are expressed as mean ± Standard deviation. Motor evoked potential, MEP; Depressor Anguli oris, DAO; First dorsalis interosseus, FDI; active motor threshold, AMT.

**MEP Amplitude DAO muscle**	**TEST MEP**	**1.0 ms**	**1.5 ms**	**2.0 ms**	**2.5 ms**	**3.0 ms**	**3.5 ms**
80% AMT	0.43±0.24	0.52±0.27	0.58±0.32	0.48±0.23	0.47±0.25	0.49±0.29	0.46±0.24
90% AMT	0.50±0.35	0.78±0.42	0.59±0.44	0.56±0.40	0.59±0.34	0.50±0.40	0.50±0.34
100% AMT	0.50±0.23	0.74±0.32	0.79±0.45	0.70±0.38	0.59±0.25	0.65±0.30	0.57±0.31
110% AMT	0.58±0.32	0.86±0.48	0.90±0.45	0.79±0.43	0.73±0.38	0.73±0.32	0.69±0.32
**MEP Amplitude FDI muscle**	**TEST MEP**	**1.0 ms**	**1.5 ms**	**2.0 ms**	**2.5 ms**	**3.0 ms**	**3.5 ms**
80% AMT	0.99±0.91	1.01±0.79	1.27±1.12	0.92±0.64	0.92±0.80	0.87±0.73	0.86±0.61
90% AMT	1.09±0.90	2.28±1.25	1.38±2.13	1.09±1.26	1.11±0.85	1.06±0.92	1.16±0.76
100% AMT	1.16±1.03	2.08±2.22	2.89±2.63	1.98±1.94	1.31±1.25	1.55±1.43	1.39±1.33
110% AMT	1.26±1.14	2.35±2.28	2.89±2.51	2.36±2.23	1.75±1.82	1.78±1.43	1.81±1.46

**MEP Amplitude Ratio DAO muscle**	**1.0 ms**	**1.5 ms**	**2.0 ms**	**2.5 ms**	**3.0 ms**	**3.5 ms**
80% AMT	1.26±0.27	1.41±0.46	1.19±0.40	1.13±0.29	1.13±0.27	1.12±0.33
90% AMT	1.24±0.32	1.71±0.53	1.22±0.37	1.17±0.32	1.22±0.31	1.04±0.27
100% AMT	1.57±0.49	1.58±0.44	1.38±0.34	1.22±0.24	1.31±0.26	1.12±0.23
110% AMT	1.57±0.84	1.68±0.76	1.41±0.55	1.33±0.50	1.35±37	1.25±0.40
**MEP Amplitude Ratio FDI muscle**	**1.0 ms**	**1.5 ms**	**2.0 ms**	**2.5 ms**	**3.0 ms**	**3.5 ms**
80% AMT	1.13±0.39	1.38±0.63	1.15±0.64	1.05±0.49	0.98±0.38	1.03±0.42
90% AMT	1.30±0.3	2.02±1.12	1.22±0.45	1.02±0.28	1.01±0.28	1.01±0.27
100% AMT	1.59±0.72	2.41±1.08	1.62±0.64	1.15±0.31	1.36±0.53	1.20±0.3
110% AMT	1.73±0.74	2.30±0.91	1.79±0.62	1.36±0.52	1.45±0.41	1.52±0.68

Table 5: data are expressed as mean ± Standard deviation. Depressor Anguli oris, DAO; First dorsalis interosseus, FDI; active motor threshold, AMT; maximum voluntary isometric contraction (MVIC)

**PS duration DAO muscle**	**10% MVIC**	**100% MVIC**
120% AMT	66.04±34.03	59.59±23.38
130% AMT	80.47±43.52	75.04±37.21
140% AMT	100.04±12.66	98.71±41.26
**PS duration FDI muscle**	**10% MVIC**	**100% MVIC**
120% AMT	60.87±35.85	54.87±32.07
130% AMT	79.01±43.29	64.94±31.11
140% AMT	92.20±42.09	91.28±53.83
